# Physical Health Care for People with Severe Mental Illness: the Attitudes, Practices, and Training Needs of Nurses in Three Asian Countries

**DOI:** 10.3390/ijerph15020343

**Published:** 2018-02-15

**Authors:** Daniel Bressington, Ashish Badnapurkar, Sachiko Inoue, Hin Yeung Ma, Wai Tong Chien, Deborah Nelson, Richard Gray

**Affiliations:** 1 School of Nursing, The Hong Kong Polytechnic University, Hung Hom, Kowloon, Hong Kong, China; hin-yeung.ma@connect.polyu.hk (H.Y.M.); wai.tong.chien@polyu.edu.hk (W.T.C.); 2 Health Services and Population Research Centre, Hamad Medical Corporation, PO Box 3050, Doha, Qatar; ABadanapurkar@hamad.qa; 3 Department of Nursing, Okayama Prefectural University, 111 Kuboki, Soja, Okayama 719-1197, Japan; sinoue@fhw.oka-pu.ac.jp; 4 Mental Health Services, Hamad Medical Corporation, PO Box 3050, Doha, Qatar; DNelson@hamad.qa; 5 Department of Nursing and Midwifery, La Trobe University, Melbourne, Victoria 3086, Australia; r.gray@latrobe.edu.au

**Keywords:** nurse attitudes, physical health care, severe mental illness, nurse education

## Abstract

People with severe mental illness (SMI) have considerable unmet physical health needs and an increased risk of early mortality. This cross-sectional survey utilized the Physical Health Attitude Scale (PHASe) to examine the attitudes, practices, and training needs of nurses towards physical health care of people with SMI in three Asian countries (Hong Kong, Japan, Qatar). Cross-country differences were explored and linear regression was used to investigate if nurses’ attitudes and confidence were associated with their level of involvement in physical health care. A total of 481 questionnaires were returned. Hong Kong nurses were less involved in physical health care than those from Japan and Qatar. Nurses’ attitudes and confidence were significant predictors of their participation in managing physical health. Compared with western countries, more nurses in this study felt that mental illness was a barrier to improving physical health. Three-quarters reported that they needed additional training in promoting cardiometabolic health. The perceived need for additional training in physical health care was held by Mental Health Nurses (MHN) irrespective of their type of nursing registration and nationality. Nurse educators and service providers should reconsider the physical health care training requirements of nurses working in mental health settings in order to improve the physical health of people with SMI.

## 1. Introduction

People with severe mental illness (SMI) have a much higher incidence of physical health problems than the general population [1,2]. The increased rates of physical illness contribute towards a reduced life expectancy of between 7 and 20 years [3,4,5]. The risk of mortality from any cause in people with a SMI is at least twice that of people without a mental illness [6,7].

Many inter-related factors negatively influence the physical health of people with SMI. These include unhealthy lifestyle and health behaviors [8], such as a lack of physical activity [9], unhealthy diet, and smoking [10]. In addition, prescribed medications, the attitudes of health care staff, difficulties in accessing care, poor uptake of health screening, and self-stigma are also likely to be contributing factors towards the poor physical health of people with SMI [1,7,11,12,13].

The need to address physical health disparities in people with SMI is clear. Despite this, the implementation of physical health promotion and screening remains sub-optimal at best [7,14]. Given that nurses working in mental health settings have the most face-to-face contacts with people with SMI, it is reasonable to assume that nurse-related factors could present barriers towards the effective implementation of physical health care [15]. Accordingly, it has been hypothesized that one reason for the poor physical health of people with SMI is the inadequate knowledge/skills and negative attitudes of Mental Health Nurses (MHN), which may result in patients’ physical health needs being overlooked [15,16,17]. Indeed, some qualitative studies conducted over the last 15 years suggest a degree of MHN pessimism towards improving the physical health of people with SMI. For example, Gray and Brown [18] interviewed 18 MHN and 15 patients about their experiences of working together to improve physical health. The authors reported that nurses did not perceive promoting physical health as a high priority and patients perceived that their physical health needs were not being adequately addressed. Similarly, Hyland et al. [19] reported that mental health case managers were pessimistic about their ability to improve the physical health of people with SMI living in the community. These negative staff attitudes are particularly concerning because they have the potential to negatively influence the diagnosis and treatment of patients with multi-morbidities [20].

Previous studies have assessed MHN attitudes and confidence in providing physical healthcare in non-Asian countries. For example, a survey using the Physical Health Attitude Scale for mental health nurses (PHASe) was conducted in England by Robson et al. [21] with 585 MHN. The authors concluded that although MHN had generally positive attitudes towards physical health care for people with SMI, they felt ill equipped to provide various aspects of care and deliver health promotion interventions. The authors also observed that higher levels of confidence and more positive attitudes were associated with greater MNH involvement in physical health care [21]. In a second study, 57 UK forensic inpatient ward staff [7] completed the PHASe. The authors reported that staff confidence in conducting basic physical health monitoring (i.e., blood pressure measurement) was high. However, a majority of participants reported that it was challenging to promote positive health behaviors with forensic service users. Along similar lines, Happell et al. [22] surveyed 600 MHN in Australia using a modified version of the PHASe. The authors reported that 60% of respondents felt they required additional training in all areas of physical health care. Training in cardiovascular disease and diabetes management were the top priorities. The most recent study using an adapted version of the PHASe was conducted in the USA [23,24]. Despite having good levels of knowledge about cardiometabolic health, only a minority of nurses reported using evidence-based physical health interventions, and a majority wanted more training.

In summary, these studies consistently show that many western MHN report the need for further education and support in order to provide evidence-based care to enhance the physical health of people with SMI.

It is unknown if MHN perceived inadequacy to address the physical health of people with SMI is a global issue. To date, research on MHN physical health care attitudes and practices has focused on the UK, USA and Australia. It cannot be assumed that earlier observations can be generalized to other parts of the world with different types of nurse training programs, cultures and traditions.

### Aim

The aim of this survey was to investigate and compare MHN practices, attitudes, perceived barriers and training needs towards physical health care for service-users with SMI in three Asian countries.

## 2. Methods

This study is a cross-sectional, self-report survey of MHN working in mental health settings in three Asian countries (Hong Kong, Japan and Qatar). We have followed the Strengthening the Reporting of Observational Studies in Epidemiology (STROBE) guidelines in reporting this study [25].

### 2.1. Study Settings and Participants

All registered nurses currently practicing in mental health inpatient or outpatient/community settings were potentially eligible to take part in the study, irrespective of their type of nursing registration (i.e., any registered nurse with/without a specialized registration as a mental health nurse). The survey was conducted in selected hospitals providing mental health services in Japan, whereas one psychiatric hospital was surveyed in both Qatar and Hong Kong. Mental Health Nurses in Hong Kong are educated following a UK-type system where they choose to specialize in mental health from the outset of their undergraduate program and upon qualification are registered as psychiatric (rather than general) nurses. The majority of the health care professional workforce in Qatar are expatriates, they have to undergo training in their home countries and register with the Qatar Council for Health Professionals as a general nurse (in Qatar there is currently no specialist register for mental health nurses) before practicing. The training of MHN in Japan follows a system similar to that in the USA and Australia, where they qualify as a registered (comprehensive) nurse before specializing in mental health after completing a post-registration mental health nursing education program.

### 2.2. Data Collection Procedures

In each country, a broadly similar data collection procedure was followed. All questionnaires were given a unique identification code to identify non-responders and hard copies of the survey were sent to eligible participants along with the study information sheets and return envelopes. A convenience-based sampling method was employed, where eligible participants from Hong Kong and Qatar were identified from lists of registered nurses provided by the hospitals and were sent survey packets via the internal post systems. Whereas in Japan, for practical reasons (due to the large number of potential participants) we selected 94 hospitals from the Web site of the Japan Psychiatric Hospitals Association (i.e., two hospitals from each of Japan’s 47 prefectures) and sent the questionnaires via post to the eligible MHN in each hospital.

The survey was sent to a total of 1253 nurses (205 in Qatar, 628 in Hong Kong and 420 in Japan). Data were collected during February 2017 in Hong Kong, from May 2016 to September 2016 in Qatar, and during January to March 2017 in Japan.

### 2.3. Measure

We used the validated PHASe questionnaire originally described by Robson and Haddad [16]. The PHASe has 28 items relating to attitudes and confidence divided into four subscales: (a) nurses’ attitudes to physical health care (10 items); (b) nurses’ confidence to provide physical health care (6 items); (c) nurses perceived barriers in delivering physical health care (7 items) and (d) nurses attitude towards smoking (5 items). Items are scored on a 5 point Likert scale ranging from 1 (strongly disagree) to 5 (strongly agree). High scores denote positive attitudes/higher confidence. To avoid set response bias, items are positively and negatively worded. The scores for the 12 negatively worded items are reversed as appropriate for analysis. The PHASe also has 14 questions on participants’ current practices delivering physical health care, which are scored on a Likert scale ranging from 1 (never) to 5 (always), and 7 questions about their perceived training needs (rated as yes, no or not sure). The psychometric properties of the tool were established in a UK study [16]. The reported Cronbach’s alpha for the four subscales was 0.86, 0.74, 0.67 and 0.61.

Before conducting the survey, we translated and validated the Japanese version of the questionnaire. First, the original English-language questionnaire was translated into Japanese by one of the research team. Next, the translation was checked by three experienced clinical nurses working in a hospital psychiatric department. After making amendments we requested a back translation by a professional translation company. We found a good level of agreement between items on the original UK version and the back-translated English language questionnaire. We also established content validity of the final Japanese version among 27 senior MHNs at five hospitals in Okayama Prefecture, Japan. The Hong Kong survey did not require translation (English is used routinely in academic/clinical settings), but content validity was checked with 13 senior MHN. In Qatar, the survey instrument also did not require language translation (English is considered the official business language), a review of the PHASe by senior nursing and clinical staff established that the questions were both understandable and relevant to local practice. The questions about staff smoking and having previous physical heath training were removed from the Japanese questionnaire after content validity checking; otherwise, the structure of the questionnaire was identical in all three countries.

After data collection was complete, we calculated the internal consistency of the PHASe questionnaire subscales for the whole sample and for each individual country using Cronbach’s Alpha. The internal consistency of the subscales was found similar to the original UK tool validation study [16]. Subscale 1 was acceptable for the three countries (0.778), Japan (0.716) and Qatar (0.770), and was good in Hong Kong (0.850). Subscale 2 showed good internal consistency over the three countries (0.849), and was acceptable for Hong Kong (0.748) and Japan (0.761), and slightly low for Qatar (0.685). The internal consistency of subscale 3 for the three countries was 0.578, with alpha coefficients of 0.577 for Japan, 0.645 for Hong Kong and 0.657 for Qatar. Subscale 4 consistency was 0.618 for the three countries, with coefficients of 0.717 in Hong Kong, 0.707 for Japan and 0.355 in Qatar. Item-total correlation analysis for the 5-item subscale 4 in Qatar revealed that the removal of question 12 would result in an improvement of Cronbach’s alpha value. However, deleting question 12 (clients should not be encouraged to give up smoking as they enough to cope with) would only result in a subscale consistency of 0.469, suggesting that this question may have been understood differently in Qatar to the other countries, and also highlighting that the scale requires further refinement for use in Qatar.

The following demographic data was requested from participants: age, gender, educational attainment, ethnicity, years of experience working in psychiatry, type of registration (adult and/or mental health), smoking status and current clinical practice setting.

### 2.4. Sample Size Calculation

As the numbers of MHN varied across the study settings we calculated our sample size based on a conservative estimate of participants required in each country to be able to conduct linear regression (using the score for nurses’ involvement with physical health care as the dependent variable) with 4 predictor variables (i.e., the four PHASe subscales) using Green’s rule of thumb i.e., *n* > 104 + m (where *n* = number of participants and *m* = number of predictor variables) [26]. Therefore, the minimum sample required for each country was 108. However, given the potential for low survey response rates we aimed to send out as many questionnaires as was practical in each country.

### 2.5. Data Analyses

Descriptive statistics were used to describe the demographic characteristics of participants. To summarize the level of agreement with individual PHASe items we reported the mean score and the percentage of participants who agreed with each statement (i.e., those scoring 4 (agree) or 5 (strongly agree) on the five-point scale). Scores for the 12 negatively worded items were reversed for calculating the subscale and total scores. There was < 0.01% missing data, these were imputed using the group median value for calculation of the relevant subscale/total score. The total scores for involvement in physical health care, total PHASe score and PHASe subscale scores were compared across each country with one-way ANOVA test. Bonferroni post-hoc tests were utilized to identify statistically significant differences between countries where ANOVA results were significant. In order to identify which individual PHASe items were answered differently in the three settings we used post-hoc Chi-square test to identify associations in the distribution of MHN that agreed/strongly agreed with each statement in the three countries. Stepwise linear regression was used to investigate predictors of MHN involvement in physical health-care in each country, with the total score for involvement in physical health care practice used as the dependent variable and PHASe subscale scores as predictor (independent) variables. We also identified any significant bivariate associations between demographic characteristics and involvement scores, and included these demographics as potential predictors in the stepwise regression models for each country as appropriate. The regression models were set to include independent variables which increased the probability of F by a minimum of 0.05 and excluded them if the increase of F was lower than 0.1. The level of significance was set at *p* < 0.05, however this was adjusted using the Bonferroni correction where multiple comparisons were carried out (by dividing 0.05 by the number of comparisons). Following Bonferroni corrections, we adopted a significance level of *p* < 0.001 for comparison of individual PHASe items and *p* < 0.0125 for comparison of PHASe subscale/total mean scores. IBM SPSS Statistics for Windows, version 23 (IBM Corp., Armonk, NY, USA)was used for all analyses.

### 2.6. Ethical Considerations

The study was approved by the institutional/hospital authority ethical review committees of each country (reference numbers: Qatar IRB16122/16; Hong Kong KW/FR-16-173 (104–10); Japan Okayama Prefectural University IRB No. 16-70). The information sheets sent out with the surveys clearly stated that participation was entirely voluntary. In order to maintain the confidentiality of participants no identifying information was included in the survey forms. The return of completed questionnaires was considered to constitute implied informed consent to participate in each setting (as stated in all participation information sheets).

## 3. Results

### 3.1. Response

In total, 481 questionnaires were returned, an average response rate of 39% for the three countries. There were differences in response rate by country (Hong Kong 23%, Japan 48%, and Qatar 67%). Overall, 42 responses to the individual PHASe questions were missing from the returned questionnaires (<0.01% of all data).

### 3.2. Demographic Characteristics

Participants’ demographic characteristics are shown in Table 1. Most participants were female (57%, *n* = 275) and in their 30 s (29%, *n* = 139). More Japanese nurses were aged 40 and over (74%, *n* = 147) when compared to those in Hong Kong (40%, *n* = 57) and Qatar (26%, *n* = 35). Nurses working in Qatar were expatriates mostly from the Middle East, North African and Asian countries. Nurses in Hong Kong and Japan were all nationals. The majority of nurses in Qatar (81%, *n* = 112) and Hong Kong (78%, *n* = 111) were educated to at least bachelor degree level, compared to one in ten (11%) Japanese nurses. 

All participants in Hong Kong were registered psychiatric nurses. In Japan, a minority (9%, *n* = 17) had a specific mental health nursing qualification and none of the participants in Qatar had this specialist registration. All participants in Qatar and Japan, but only 17% (*n* = 24) in Hong Kong, were registered general nurses. Most participants had over 5 year’s clinical experience working in a mental health setting. Nurses in Hong Kong had less experience (38% having 0–5 years’ experience) compared with those in Japan and Qatar (5% and 1% respectively). Most nurses (82%, *n* = 394) were currently working in inpatient psychiatric units, although a quarter of nurses (27%, *n* = 39) in Hong Kong were working in community settings. Few nurses (5%, *n* = 22) had received post-registration training on physical health care for people with SMI. Few participants (3%, *n* = 12) reported that they were current smokers (noting that smoking data were not collected from Japan).

### 3.3. Involvement in Physical Health Practice

Table 2 shows the current physical health care practice of respondents. The majority of nurses stated that they would always or very often check whether a patient’s physical health was assessed when first providing nursing care. The routine and hands-on aspects of physical health care, such as monitoring blood pressure and assisting with personal hygiene were also carried out by about three quarters (71–88%) of respondents. Similarly, assessing bowel habits was also always or very often performed by more than 60% of MHN in all countries.

Hong Kong nurses were less involved than those in Qatar and Japan in some other aspects of physical health care. Specifically, only 19% (*n* = 27) of Hong Kong MHN would always/very often check that a patient was registered with a family doctor, compared with 85% (*n* = 170) in Japan and 55% (*n* = 77) in Qatar. Similarly, regularly weighing service-users was conducted always or very often by 51% (*n* = 73) of participants in Hong Kong, compared to 80% (*n* = 110) in Qatar and 73% (*n* = 146) in Japan.

The provision of health promotion advice (e.g., healthy eating, exercising regularly, stopping smoking, dental health and helping with weight management) was also far less common in Hong Kong than in the other two countries. Less than a third of MHN in all three countries were always/very often involved in giving contraceptive advice or ensuring service-users had a regular eye examination.

A comparison of MHN involvement in physical healthcare total mean scores across the three countries (using one-way ANOVA test) highlighted significant differences between groups (*p* < 0.001). Bonferroni post-hoc test revealed that Hong Kong MHN had significantly lower mean scores than those from Japan (MD = −6.64, 95%CI −8.76 to −4.52, *p* < 0.001) and Qatar (MD = −8.27, 95%CI −10.59 to −5.96, *p* < 0.001). Independent sample t-test showed that MHN who were Registered General Nurses (M = 52.70, SD = 8.07) had significantly greater involvement in physical healthcare than participants with only a Mental Health (psychiatric) registration (M = 46.40, SD = 9.01), *t* (479) = 7.17, *p* < 0.001).

### 3.4. Physical Health Attitude Scale for Mental Health Nurses Total and Subscale Scores

Table 3 shows a comparison of the PHASe subscale and total mean scores across the three countries. One-way ANOVA tests show significant differences on subscales one (attitudes towards involvement in physical health care; *p* = 0.012), two (Nurses’ confidence in delivering physical health care; *p* < 0.001) and four (Nurses’ attitudes towards smoking; *p* < 0.001). Bonferroni post-hoc tests demonstrated that Japanese MHN had significantly less positive attitudes towards involvement in physical health care than MHN in Qatar (MD = −1.61, 95%CI −2.98 to −0.24, *p* = 0.015). Japanese MHN also had lower confidence in delivering physical healthcare than those in both Hong Kong (MD = −4.58, 95%CI −5.39 to −3.76, *p* < 0.001) and Qatar (MD = −5.98, 95%CI −6.80 to −5.16, *p* < 0.001). The post-hoc tests showed that MHN from Qatar had less positive attitudes towards promoting smoking cessation that those in Hong Kong (MD = −1.39, 95%CI −2.28 to −0.49, *p* < 0.0001) and Japan (MD = −1.58, 95%CI −2.41 to −0.74). There is also a statistically significant difference in total mean PHASe scores between the three countries (*p* < 0.001). Bonferroni post-hoc tests revealed that MHN from Japan had significantly lower total scores than those in Hong Kong (MD = −3.81, 95%CI −6.33 to −1.30, *p* = 0.001) and Qatar (MD = −4.70, 95%CI −7.24 to −2.15, *p* < 0.001).

### 3.5. Perceived Training Needs

Table 4 shows the perceived training needs of MHN. More than half (59% to 82%) of all respondents perceived that they needed information/training on all the areas of physical healthcare listed in the PHASe (except “how to discuss reproductive health issues with patients” perceived as a need by just under a half (49%, *n* = 70) of nurses from Hong Kong and a third (33%, *n* = 66) of nurses in Japan). Almost three-quarters (67–78%) of MHN from all three countries perceived that they needed additional training on cardiovascular health and diabetes management.

### 3.6. Responses to Individual Physical Health Attitude Scale for Mental Health Nurses Items

#### 3.6.1. Attitudes towards Involvement and Confidence in Delivering Physical Health Care

Four of the individual attitude items were found to be significantly different between countries. Fewer Japanese nurses (56%, *n* = 111) than those in Qatar (89%, *n* = 123) or Hong Kong (74%, *n* = 106) believed that giving nutritional advice was part of their role (X^2^ [2, N = 481] = 45.73, *p* < 0.001). Whereas more MHN from Hong Kong (67%, *n* = 96) than those from Qatar (27%, *n* = 37) or Japan (34%, *n* = 67) perceived that they should give contraceptive advice to service-users (X^2^ [2, N = 481] = 55.76, *p* < 0.001). There were also significant differences in MHN attitudes towards educating male service-users about testicular self- examination (X^2^ [2, N = 479] = 48.74, *p* < 0.001), with fewer MHN in Japan (17%, *n* = 33) than in Hong Kong (38%, *n* = 54) or Qatar (51%, *n* = 71) agreeing that this should be part of their role. MHN from Qatar (73%, *n* = 100) were more likely to agree that their role involved educating female patients about the importance of breast self-examination (X^2^ [2, N = 480] = 30.51, *p* < 0.001) than those from Hong Kong (44%, *n* = 63) or Japan (46%, *n* = 92). The confidence-related individual items were all significantly different across countries, with Japanese MHN typically reporting feeling less confident than nurses in the other settings.

#### 3.6.2. Perceived Barriers

Perceived barriers to physical healthcare delivery were identified by MHN in all countries. The most frequently cited/agreed barriers were: MHN workloads prevented them from engaging in physical health promotion (37–45%), informing patients of the effects of medications on physical health would increase non-adherence (46–48%), patients are not motivated to exercise (29–41%) and patients are not interested in improving their physical health (32–48%). Japanese MHN (17%, *n* = 33) were found to be significantly less likely than MHN in Hong Kong (62%, *n* = 88) and Qatar (60%, *n* = 82) to agree that it could be difficult to get patients to follow advice about diet (X^2^ [2, N = 481] = 98.35, *p* < 0.001). However, significantly fewer MHN in Hong Kong (29%, *n* = 42) agreed that patients’ worries about physical health are due to their mental illness (X^2^ [2, N = 480] = 88.10, *p* < 0.001) compared with those in Qatar (56%, *n* = 77) or Japan (80%, *n* = 160).

#### 3.6.3. Attitudes towards Smoking

In terms of MHN attitudes towards smoking, most participants agreed that staff should be banned from smoking on healthcare premises (61–75%). Although a minority of MHN across all three countries (3–18%) believed that service-users should be given cigarettes to help achieve therapeutic goals, the MHN in Qatar were most likely to agree on this statement (X^2^ [2, N = 480] = 33.58, *p* < 0.001). The majority of MHN in both Japan (74%, *n* = 147) and Hong Kong (69%, *n* = 98) stated that service-users should be banned from smoking in hospital premises, whereas significantly less (41%, *n* = 56) of MHN in Qatar supported this position (X^2^ [2, N = 480] = 33.58, *p* < 0.001). 

### 3.7. Regression Models

Stepwise linear regression was conducted in order to identify which PHASe subscales and associated demographic variables were significant predictors of MHN involvement in physical health care in each country. Collinearity tests confirmed the independency of predictor variables (tolerance range = 0.74 to 0.98, VIF range = 1.01 to 1.34) and assumption of independent errors of residuals was met (Durbin-Watson values ranged from 1.72 to 2.02) in all three models.

The Qatar model (F (2, 135) = 14.78, *p* < 0.001, *R*^2^ = 0.18, R^2^_Adjusted_ = 0.17) showed that subscales 1 and 2 (MHN attitudes towards physical healthcare and MHN confidence in providing physical healthcare) were significant predictors of the level of MHN involvement in the physical health care of their clients, accounting for 17% of the variance. Similarly, the Hong Kong model also showed that subscales 1 and 2 accounted for 25% of the variance in mean scores for MHN involvement in physical health care (F (2, 142) = 24.42, *p* < 0.001, *R*^2^ = 0.26, *R*^2^_Adjusted_ = 0.25). The Japanese model showed that PHASe subscales 1, 2, 4 (MHN attitudes towards smoking) and age (≥40 years) accounted for 25% of variance in involvement scores (F (4, 194) = 17.81, *p* < 0.001, *R*^2^ = 0.27, *R*^2^_Adjusted_ = 0.25). Beta-coefficients and *p*-values of the three models are shown in Table 5.

## 4. Discussion

This survey aimed to describe MHN attitudes, perceived barriers and training needs related to physical health care and explore potential predictors of their involvement in physical healthcare practice. The results showed that MHN attitudes and confidence were found to significantly predict the extent of their participation in their service-users’ physical health care. This finding is broadly consistent with previous studies using the PHASe in the UK [21] and the USA [23]. The Japanese regression model included subscale four (attitudes towards smoking) and older age (≥40 years) as significant explanatory variables in addition to subscales one and two. The relationship between age and engagement in physical health care practices is worthy of further investigation as this was also previously identified in the UK PHASe study [21].

General nurses in the current study were found to have significantly greater involvement in physical healthcare than those with only a mental health (or psychiatric nurse) registration. This observation is consistent with Robson et al. [21] in the UK, and may further reinforce the hypothesis that pre-registration nurse training specialized in mental health may not adequately prepare a MHN for promoting and managing the physical health conditions that are extremely prevalent in people with SMI. That said, as the majority of nurses without a general (adult) registration were based in Hong Kong, the local clinical practice context could account for the lack of involvement, rather than their type of nursing registration. Therefore, in addition to MHN education, consideration of the organizational dynamics that may help or hinder the implementation of physical health care or promotion is essential. As suggested by authors of previous studies, provision of time and resources, and the empowerment of staff are required to effectively integrate evidence-based interventions for physical health into routine mental health nursing practice [23,27]. Although MHN in Hong Kong did not score significantly differently from those in the other two countries on the perceived barriers subscale, they were significantly more likely to agree that their workload prevented them from engaging in physical health promotion with service-users.

Nurses in this study had slightly less favorable attitudes towards involvement in the physical health care of people with SMI (as measured by 10 PHASe items), when compared with the UK studies reporting a subscale mean of 36.62 in a mental health NHS trust [21] and 39.86 on a forensic psychiatric unit [7]. MHN in this study had generally positive attitudes towards involvement in aspects of care for physical health problems most commonly seen in people with SMI. For example, the MHN had comparable levels of agreement (65–73%) with those in the UK (75% in Robson and Haddad, [21]; 84% in Haddad et al. [7]) that providing advice to prevent heart disease was part of their role. However, this was less than that reported in the USA (93%) [23]. Similarly, the majority of MHN in this study believed that they should help service-users manage their weight (76–86%) and give nutritional advice (56–89%), showing similar results to those reported from the UK [7,21] and the USA [23].

Compared with the findings from the UK [21] and the USA [23], more nurses in this study felt that mental illness was a barrier to improving service-users’ physical health. For example, a substantial proportion of the participants agreed that service-users with mental illness were not interested in improving their physical health and were not motivated to exercise. In addition, almost a third of the MHN in Hong Kong, more than half of those from Qatar, and 80% of those in Japan perceived that patient’s physical health worries were mostly due to their mental illness. As serious physical health problems are common in people with SMI, this negative attitude is problematic because it is highly likely to result in MHN overlooking the physical health needs of their service-users. The misattribution of physical symptoms to mental illness has been labelled as “diagnostic overshadowing” [28]. The process of diagnostic overshadowing may be partially responsible for the physical health disparities observed in people with SMI. Previous studies showed that people with mental illness who had serious physical illnesses presenting to general hospitals were less likely to be admitted to hospital and receive appropriate treatment, when compared to those without a mental illness [29,30,31].

The Japanese nurses in this study reported feeling less confident in providing all aspects of physical health care, compared to those in Hong Kong and Qatar. Given that all the Japanese nurses in this study are registered general nurses, this finding seems dissonant with the hypothesis that pre-registration general nurse training is more likely to result in nurses having high levels of confidence in physical health care. However, it is possible that these low confidence levels are enshrined in Japanese culture and nursing practice, where modesty is seen as a virtue and nurses may be traditionally expected to perform tasks under the close supervision of medical colleagues rather than as independent practitioners [32,33,34,35]. Indeed, previous studies, which directly compare Japanese nurses’ confidence in performing nursing interventions for diabetes and cancer patients with those in nurses from Western countries, have also reported that their levels of confidence were comparatively low [36,37].

Participants reported many perceived physical health care training needs. Around two-thirds of them wanted more training in all areas mentioned in the survey (except reproductive health issues). The MHN from Japan expressed the greatest need for training relating to providing advice on safe exercise (81% versus approximately 60% in both Qatar and Hong Kong). These findings concur with results reported from the UK [14,16,21], the USA [23,24] and Australia [22]. The overall picture is therefore of a high level of perceived need for additional training in order to provide effective holistic care for people with SMI.

## 5. Study Limitations

This study has several limitations that mainly arose from conducting the study in three different countries. We utilized a questionnaire with satisfactory reliability and validity in the UK, but requiring translation in Japan and content validity checking in all three countries. However, the processes of establishing content validity varied slightly across the three countries and this might have negatively affected the psychometric properties of the survey instrument. The overall internal consistencies of the tool and its subscales were found to be similar to those reported in the original UK study, but the alpha coefficient for subscale 4 (smoking) was unacceptably low in Qatar. We also used convenience sampling in the three countries, and in different ways, which might have resulted in selection bias. The response rates were also quite different across settings (70% in Qatar, 23% in Hong Kong and 48% in Japan), which might result in risks of nonresponse bias, and thus reduce the representativeness of the sample and generalizability of the findings.

## 6. Implications and Conclusions

The findings of this study indicate that Asian nurses working in mental health settings had generally positive attitudes towards providing physical health care. However, there was a lack of confidence and involvement in relevant interventions, and the majority identified a need for further physical health care education specific to the needs of people with mental illness. Compared with the findings from western countries, more nurses in this study felt that mental illness was a barrier to improving service-users’ physical health and many attributed patient’s physical health worries to their mental illness. Therefore, physical health care education programs in the region should have a specific focus on exploring and modifying nurse attitudes that may result in them overlooking the physical health needs of people with severe mental illness. In addition, educational interventions should aim to improve the confidence levels of nurses, perhaps by providing knowledge and supervised skills practice for managing the most prevalent physical health problems of this patient population. Given the limitations of this study, subsequent cross-sectional studies should aim to recruit a large randomly selected sample of nurses in order to improve the generalizability of the findings and consider using mixed methods (i.e., quantitative survey with qualitative interviews) to explore issues in greater depth. Future experimental studies could consider testing the effects of physical health care education programs on both nurse and patient-related outcomes.

Our results, and those of previous studies, seem to suggest that mental health nurses perceive a need for additional physical health care education irrespective of their type of nursing registration, nationality and location of clinical practice. Nurse educators and service providers should therefore reconsider the physical health care educational requirements of nurses working in mental health settings in order that they are better equipped to manage the co-morbidities that are very common in people with mental illness.

## Figures and Tables

**Table 1:** Demographic information.

Demographic Variable	Qatar N (%)	Japan N (%)	Hong Kong N (%)	Total N (%)
Number of participants	138 (100%)	200 (100%)	143 (100%)	481 (100%)
Gender				
Male	62 (45%)	74 (37%)	67 (47%)	203 (42%)
Female	73 (53%)	126 (63%)	76 (53%)	275 (57%)
Unspecified	3 (2%)	-	-	3 (1%)
Age				
29 years or younger	24 (17%)	7 (4%)	54 (32%)	76 (16%)
30–39 years	53 (38%)	45 (23%)	41 (29%)	139 (29%)
40–49 years	27 (20%)	75 (38%)	18 (13%)	120 (25%)
50 or over	8 (6%)	72 (36%)	39 (27%)	119 (25%)
Unspecified	26 (19%)	1 (0.5%)	-	27 (6%)
Ethnicity				
Middle East and North Africa	47 (34%)	-	-	47 (10%)
East Asia	31 (23%)	200 (100%)	143 (100%)	374 (78%)
South Asia	21 (15%)	-	-	21 (4%)
Sub-Saharan Africa	2 (1%)	-	-	2 (1%)
Unspecified	37 (27%)	-	-	37 (8%)
Years of experience in Psychiatry				
0–5 years	1 (1%)	10 (5%)	45(38%)	65 (14%)
6–15 years	67 (49%)	72 (36%)	26 (18%)	165 (34%)
16–25 years	25 (18%)	74 (37%)	22 (15%)	121 (25%)
More than 25 years	10 (7%)	38 (19%)	41 (29%)	89 (19%)
Unspecified	35 (25%)	6 (3%)	-	41 (9%)
Highest Academic Qualification				
Certificate	-	-	17 (12%)	17 (4%)
Diploma	20 (15%)	162 (81%)	15 (11%)	197 (41%)
Associate degree	-	13 (7%)	-	13 (3%)
Bachelor degree	101 (73%)	12 (6%)	70 (49%)	183 (38%)
Post graduate diploma and above	11 (8%)	10 (5%)	41 (29%)	62 (14%)
Unspecified	6 (4%)	3 (2%)	-	9 (2%)
Registered Mental Health Nurse				
Yes	-	17 (9%)	143 (100%)	160 (33%)
Registered General Nurse				
Yes	138 (100%)	200 (100%)	24 (17%)	362 (75%)
Current practicing Unit				
Inpatient	104 (75%)	200 (100%)	90 (63%)	394 (82%)
Out patient	8 (6%)	-	14 (10%)	22 (5%)
Community mental health Psychiatry	13 (9%)	-	39 (27%)	52 (11%)
Other	5 (4%)	-	-	5 (1%)
Unspecified	8 (6%)	-	-	8 (2%)
Additional physical health training				
Yes	21 (15%)	-	1 (1%)	22 (5%)
No	110 (80%)	-	142 (99%)	252 (52%)
Unspecified	4 (3%)	-	-	207 (43%)
Current smoker				
Yes	10 (7%)	-	2 (1%)	12 (3%)
No	124 (90%)	-	141 (99%)	265 (55%)
Unspecified	4 (3%)	200 (100%)	-	204 (42%)

**Table 2:** Participants’ current practice in physical health care and cross-country comparison.

	Qatar		Hong Kong		Japan	
	Always/Very Often N (%)	Median (Q_1_–Q_3_)	Always/Very Often N (%)	Median (Q_1_–Q_3_)	Always/Very Often N (%)	Median (Q_1_–Q_3_)
**My current practice involves** Checking if patients have had their general physical health assessed when they first come into contact with our service	106/138 (77)	5 (4–5)	85/143 (59)	4 (3–5)	160/200 (80)	4 (4–4)
Checking if the patients I work with are registered with a GP (family or primary care doctor)	77/135 (55)	4 (3–5)	27/143 (19)	2 (2–3)	170/200 (85)	4 (4–4)
Assisting patients to attend to their personal hygiene	119/137 (86)	5 (4–5)	102/143 (71)	4 (3–5)	175/200 (88)	4 (4–4)
Monitoring patient’s blood-pressure	125/138 (91)	5 (5–5)	101/143 (71)	4 (3–5)	177/200 (89)	4 (4–4)
Giving patients advice on the benefits of exercising regularly	110/138 (80)	5 (4–5)	72/143 (50)	4 (3–4)	153/200 (77)	4 (4–4)
Helping patients manage their weight	91/138 (66)	4 (3–5)	64/143 (45)	3 (3–4)	169/200 (85)	4 (4–4)
Giving patients advice on how to eat healthily	101/137 (73)	5 (3–5)	62/143 (43)	3 (3–4)	159/200 (80)	4 (4–4)
Assessing patients’ bowel habits	85/138 (62)	4 (3–5)	99/143 (69)	4 (3–5)	190/200 (95)	4 (4–4)
Giving patients advice on dental health	67/135 (49)	3 (3–5)	22/143 (15)	2 (2–3)	135/199 (68)	4 (3–4)
Testing patients for glucose abnormalities (e.g., checking glucose in urine/checking patients BM)	87/137 (63)	4 (3–5)	72/143 (50)	4 (3–5)	152/200 (76)	4 (4–4)
Weighing patients routinely throughout their contact with our service	110/138 (80)	5 (4–5)	73/143 (51)	4 (3–4)	146/200 (73)	4 (3–4)
Helping patients to stop smoking	83/138 (60)	4 (3–5)	46/143 (32)	3 (2–4)	142/199 (71)	4 (3–4)
Giving patients contraceptive advice	25/137 (18)	2 (1–3)	30/143 (21)	2 (2–3)	65/199 (32)	3 (3–4)
Ensuring patients have their eyesight assessed regularly	45/138 (33)	3 (2–4)	18/143 (13)	2 (2–3)	46/198 (23)	3 (3–3)
**Total Practice Involvement Score**	**Qatar** **Mean (SD)**		**Hong Kong** **Mean (SD)**		**Japan** **Mean (SD)**	***F* test ^#^**
	54.28 (9.54)		46.01 (9.35)		52.65 (5.57)	42.98*

**#** one-way ANOVA; * significant at *p* < 0.05.

**Table 3:** Comparison of Physical Health Attitude Scale for mental health nurses (PHASe)subscale mean scores and total score.

Subscale	Possible Score	Qatar	Hong Kong	Japan	Total	F, *p*
		Mean (SD)	Mean (SD)	Mean (SD)	Mean (SD)	
1. Nurses’ attitude to involvement in physical health care	50	35.50 (5.45)	34.03 (5.83)	33.89 (4.37)	34.39 (5.20)	4.47, 0.012 *
2. Nurses’ confidence in developing physical health care	30	24.69 (2.71)	23.29 (2.89)	18.71 (3.46)	21.79 (4.07)	176.68, < 0.001 *
3. Nurses’ perceived barriers to physical health-care delivery	35	19.71 (4.32)	20.31 (4.37)	21.02 (3.54)	20.43 (4.06)	4.41, 0.013
4. Nurses’ attitude to smoking	25	18.00 (3.07)	19.38 (3.23)	19.58 (3.11)	19.07 (3.20)	11.34, < 0.001 *
Total Score	140	97.89 (8.93)	97.01 (11.6)	93.20 (8.29)	95.68 (9.78)	11.81, < 0.001 *

* significant after Bonferroni adjustment (*p* < 0.0125) # one–way ANOVA test of group differences in mean scores across countries.

**Table 4:** Respondents’ perceived training needs.

Training Needs	Qatar	Hong Kong	Japan
	Yes/N (%)	Yes/N (%)	Yes/N (%)
**I would like more training on** How to care for mental health patients with diabetes	93/138 (67)	102/143 (71)	155/200 (78)
How to help patients manage their cardiovascular health	100/137 (73)	108/143 (76)	151/199 (76)
Interventions to help patients eat more healthily	81/136 (59)	89/143 (62)	150/199 (75)
How to help patients exercise safely and effectively	85/138 (62)	87/143 (61)	163/199 (82)
How to help patients stop smoking	100/137 (73)	87/143 (61)	127/200 (64)
Interventions to help patients manage their weight	89/138 (65)	90/143 (63)	155/200 (78)
How to discuss reproductive health issues with patients	85/137 (62)	70/143 (49)	66 /198 (33)

**Table 5:** Results from stepwise linear regression models–PHASe subscale and other significant predictors of MHN involvement in physical health-care practice.

Country	*b*	SE	Beta	*p*	Adjusted *R*^2^ (*p*)
**Qatar**					
Subscale 1	0.38	0.14	0.22	0.008	
Subscale 2	1.06	0.29	0.30	<0.001	
					0.167 (< 0.001)
**Hong Kong**					
Subscale 1	0.49	0.14	0.31	<0.001	
Subscale 2	0.90	0.27	0.28	0.001	
					0.248 (< 0.001)
**Japan**					
Subscale 1	0.38	0.08	0.30	<0.001	
Subscale 2	0.38	0.10	0.24	<0.001	
Subscale 4	0.26	0.12	0.15	0.024	
Age *^§^*	1.8	0.80	0.14	0.024	
					0.253 (< 0.001)

b = Unstandardized regression coefficient; Beta = Standardized regression coefficient; SE = Standard Error; Subscale 1: Nurses’ attitudes towards involvement in physical health care; Subscale 2: Nurses’ confidence in delivering physical healthcare; Subscale 4: Nurses’ attitudes towards smoking; ^§^ Greater involvement in physical health care associated with older age (≥40 years).
